# Investigation of the last two centuries sedimentation dynamics in high-altitude lakes of Southern Carpathians, Romania

**DOI:** 10.1038/s41598-024-51812-2

**Published:** 2024-01-16

**Authors:** Robert-Cs. Begy, Codrin-F. Savin, János Korponai, Enikő Magyari, Tibor Kovács

**Affiliations:** 1 Faculty of Environmental Science and Engineering, Babes-Bolyai University, 400084 Cluj-Napoca, Romania; 2 Interdisciplinary Research Institute On Bio-Nano-Sciences, Babes-Bolyai University, 400271 Cluj-Napoca, Romania; 3 Department of Water Supply and Sewerage, University of Public Service, Bajcsy-Zsilinszky utca 12–14, Baja, 6500 Hungary; 4 HUN-REN-MTM-ELTE Research Group for Palaeontology, Ludovika Tér 2, Budapest, 1083 Hungary; 5 Department of Environmental and Landscape Geography, Eötvös Loránd University, Pázmány Péter Stny 1/C, Budapest, 1117 Hungary; 6 Institute of Radiochemistry and Radioecology; Research Centre for Biochemical, Environmental and Chemical Engineering, University of Pannonia, Veszprém, 8200 Hungary

**Keywords:** Environmental impact, Sedimentology, Ecosystem ecology

## Abstract

This study investigates the last two centuries sedimentation dynamics in four high-altitude lakes located in Southern Carpathians, Romania. Furthermore, a novel approach is proposed for identifying the anthropic or natural underlying causes, by comparison of the acceleration of the change in sedimentation rate with a baseline growth rate trend provided by an isolated peat bog. The high-resolution chronologies were developed using the ^210^Pb dating technique and the CRS model. ^137^Cs alternative time-marker validated the age-depth models and reassured the quality of the results. The results indicated several short-interval high sedimentation events within the lake cores, yielding up to five times the average rate for the investigated period. The cause of the high sedimentation episodes was generally attributed to anthropic activities (primarily road construction) and extreme natural events. A first-order derivative equation was employed to plot the acceleration in the sedimentation rate of the lakes with the peat bog baseline. The discrepancies between the acceleration trends highlighted significant deviations from the natural variation tendencies and provided preliminary data regarding the underlying causes of the intense sedimentation periods.

## Introduction

Lake sediments represent key natural archives recording shifts and variations in the watershed erosional processes and sediment delivery of allochthonous material to the lake^1^. The temporal variability in sediment delivery to the lake, occurring at the catchment level, can be assessed by investigating the sedimentation trends in lake cores, as they provide relatively continuous and high temporal resolution deposition history^1^. A growing number of scientific studies report major degradation of lake ecosystems related to increased sedimentation over past decades^2–5^, with estimates indicating a global average annual water storage loss between 0.5 and 1%, directly caused by this process^6,7^.

Recognizing the global recorded increases in erosion and sedimentation trends of lacustrine depositional systems, as well as the subsequent environmental alterations and social risks associated, the investigation of the underlying causes of these processes proves compulsory. A specific challenge in the analysis of sedimentation rate variability consists in distinguishing natural stressors from anthropic influences, and their contribution to the increased sedimentation rates (SRs). The variability of lakes SRs is time-scale dependent and can be divided into^8^: (I) Short-term variations, generally attributable to catchment level influences such as flood variability (which may be also influenced by anthropic activities) and (II) Long-term (years to decades/centuries), related to both anthropic interventions and climate variability^9^. Besides extreme and massive natural events (e.g. landslides), that may induce a sudden, short-term significant increase in the lake SRs, the impact of climate variability may be recorded as a slight increase in the slope of the trend, extended over a longer timeframe. Contrary, anthropic interventions (e.g. construction of a road/dam) can induce peaks in SRs, during which soil erosion and delivery to the lake were drastically modified. Additionally, the effects of land cover changes may have long-lasting effects on the trends of the SRs.

A potential approach that can assist the process of defining and establishing the underlying responsible factor (climatic/anthropic) associated with a high SR event/trend in a lake system is the comparison with a baseline trend. A baseline level can be obtained by constructing a high-resolution trend of the sediment dynamics of an isolated, anthropically unaltered lacustrine sediment core, thus exclusively revealing the natural perturbances on the SRs. By comparison and subtraction of the baseline level, an assessment of the SRs trends in adjacent lakes can be performed, and the residual differences may be attributable to anthropic factors. By the same reasoning, peat bog cores may have similar applicability, due to their intrinsic sensitivity to rainfall (water table level) and temperature. This novel approach is proposed in the present work and has not yet been addressed by other authors in the scientific literature.

On a centennial time scale, the study of sedimentary processes and trends of lakes can be achieved by radiometric ^210^Pb dating. Proposed by Goldberg^10^ in 1963 and first applied in lacustrine sediments by Krishnaswamy et al., in 1971^11^, the technique is based on the decay curve of atmospherically deposited ^210^Pb, found in excess with respect to its parent radionuclide, ^226^Ra, in sediment cores. The high temporal resolution chronologies of recent lake deposits enable the comprehensive study of the SRs trends and high sedimentation events recorded in the analyzed cores^12^. The temporal applicability of the ^210^Pb technique is most relevant due to its coverage of both the industrial revolution and the Modern Warm Period (MWP), during which significant natural and anthropic changes occurred globally. The use of ^137^Cs alternative time-marker further confirms the validity of the dating models and reassures the quality of the results.

The present study aims to first assess the past two centuries’ sedimentation dynamics in four high-altitude lakes located in Romania and to further identify the underlying natural or anthropic causes of high sedimentation events, by proposing an approach based on intercomparison with a baseline level provided by a high-altitude anthropically unaltered lake and peat bog.

## Materials and methods

### Study site and sampling

The following high-altitude lakes, located in the Southern Carpathians, Romania were sampled:

Zănoaga (ZA) glacial lake (45°20′47.35"N; 22°49′22.11"E) situated in Retezat Mts., with a surface of 6 ha, maximum depth of 29 m, an altitude of 1997 m a.s.l., and a catchment area of 56.6 ha The ZA lake is included in Retezat National Park, which was designated as a protected area since 1979 by UNESCO “Man and the Biosphere” Committee.

Muntinului (MUN) glacial lake (45°21′59.14"N; 23°39′12.80"E), with an area of about 0.15 ha and an elevation of 1994 m a.s.l., located in Latoriței Mts. Of Parâng Massif, 200 m from the Transalpina national road.

Latoriței (LIL) glacial lake (45°22′1.48"N; 23°42′3.75"E) with a surface of 0.81 ha, catchment area of 0.57 km^2^, maximum depth of 1.5 m situated at 1630 m a.s.l. in the Latoriței Mts. (part of Parâng Massif). The lake has been declared a nature reserve since the year 2000. Both MUN and LIL are located nearby (~ 3.7 km apart), and both are close to the national Transalpina road (DN67C) constructed between 1934 and 1939.

Bâlea (BA), the largest glacial lake in Southern Carpathians (45°36′11.45"N; 24°37′1.48"E) at an altitude of 2034 m a.s.l. in Făgăraș Mountains, with a surface area of 4.7 ha, maximum depth of 11.4 m and a catchment area of 68.16 ha. The lake is situated above the tree line, close to the Transfăgărășan national road (DN7C), and has been declared a natural reserve since 1932. A meteorological station was established at the lake location in 1980.

The sampling was performed using a flag-gravity corer (inner diameter of 10.6 cm), the depth of the sediment cores ranged between 18.5 and 57.5 cm and were sub-sampled in 1 to 2 cm segments. The selected lakes are located in natural environments, with limited anthropic intervention, due to the high altitude of the sites. Grazing and/or agricultural activities are highly improbable, while road construction and hydro technical constructions are the only documented anthropic interventions in the area.

**Figure 1:**
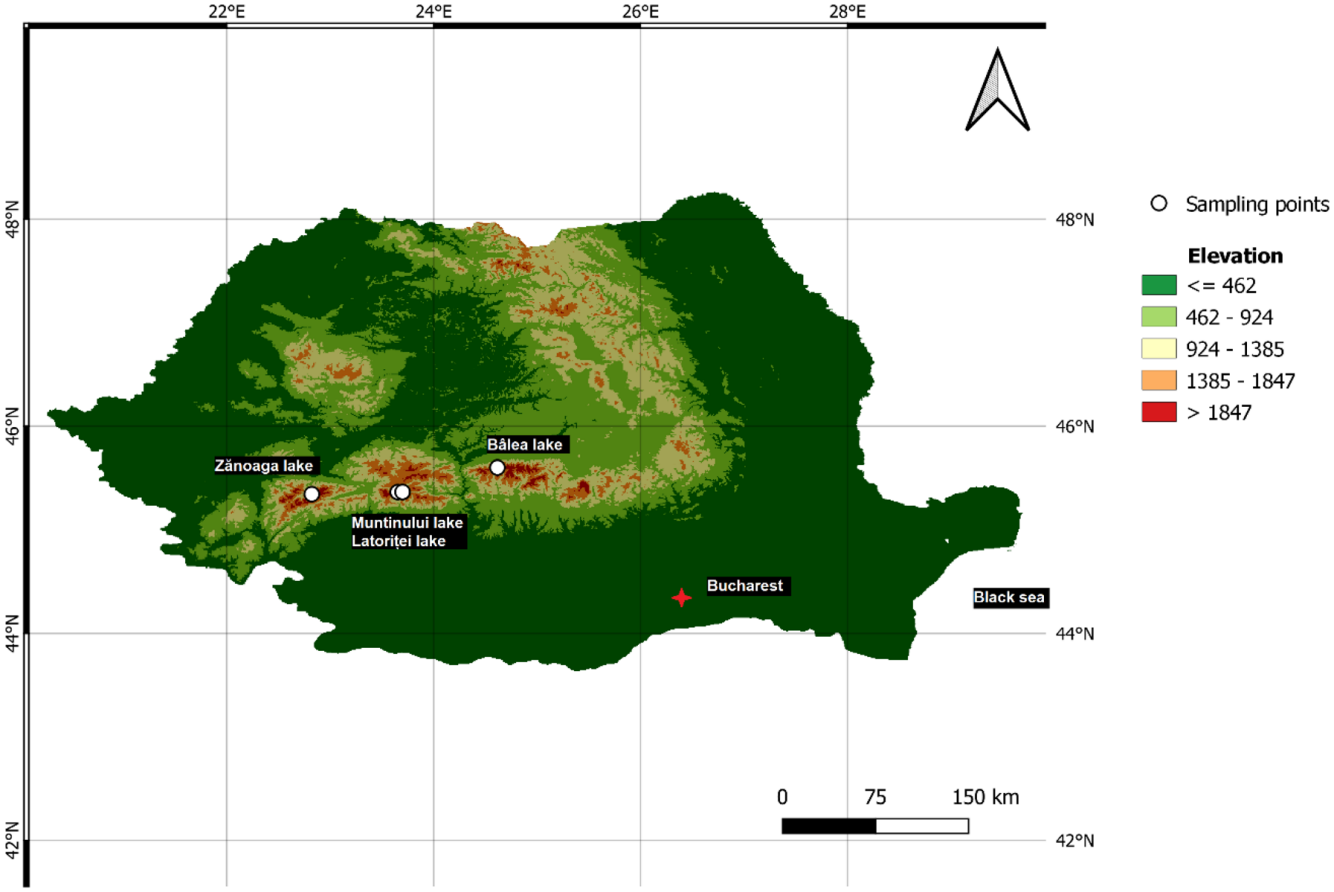
The elevation map of Romania (Europe) highlighting the sampling points of four high-altitude lakes.

A peat bog core (LIP) of 48 cm depth was sampled in the immediate proximity of the Latoriței lake (45°22′8.91″N, 23°42′5.30″E), using a Wardenaar-type corer with 10 × 10 cm surface. The Latoriței Peat bog (LIP) is a ”raised” *Sphagnum*-dominated, ombrotrophic ecosystem, the growth rate is solely controlled by atmospheric precipitations and temperature. Due to the position and altitude of the peat bog, it can be considered that the anthropic activities in the area are minimal, and the variations in peat development are exclusively due to climatic factors. Bulk wet/dry density and water content were determined gravimetrically for all the samples, by oven-drying at 105 °C until constant weight. The organic and inorganic matter fractions were determined by approaching the Loss on Ignition method, burning 1 g of sample for 4 h at 750 °C. The location and elevation features of the study area are presented in Fig. 1.

### Radionuclide measurements and dating model

The chronologies for the lake and peat bog cores were established using excess ^210^Pb. The oven-dried subsamples were ground to a fine powder and transferred into 50 mm length, 10 mm diameter High-density polyethylene vials, and further stored for a minimum of 28 days to assure the secular equilibrium between ^226^Ra and its decay products.

The radioisotopic measurements (^210^Pb, ^226^Ra, and ^137^Cs) were performed employing an ORTEC High Purity Germanium (HPGe) GWL Co-Axial Well-Type gamma spectrometer, with a resolution of 2.08 keV for 1.33 MeV ^60^Co and 1.1 keV for 122 keV ^57^Co gamma lines. The relative efficiency of the detector was 34.2% and a measurement time greater than 48 h was considered, to ensure low uncertainties. The specific activity of the radionuclides was determined through the 46.5 keV (^210^Pb) and 661 keV (^137^Cs) gamma lines, while ^226^Ra was indirectly determined by the short-lived daughters of ^222^Rn (^214^Pb at 295 keV and 351 keV; ^214^Bi at 609 keV). The activity concentrations were calculated using the relative method and the IAEA -312; IAEA -327 and IAEA -385 standards.

As the activity of ^210^Pb is decreasing exponentially with depth, in the lower strata of the cores, alpha spectrometry proved to be a more reliable technique that provided decreased limits of detection. Following this technique, the total ^210^Pb activity was indirectly determined through ^210^Po, with which it reached secular equilibrium after 2 years. The subsamples were chemically treated by acidic digestion, and the ^210^Po fraction was further spontaneously deposited on high Ni-content stainless steel disks. The chemical yield was determined isotopically through the addition of a ^209^Po tracer, the technique is further explained in detail by Begy et al., 2018^13^. Following the deposition, the disks were measured using an ORTEC Soloist alpha spectrometric system with Ultra ENS-U900 PIPS detectors (active surface of 900 mm^2^) with a resolution of 19 keV. The data acquisition was performed employing an ASPEC-92 data acquisition system.

The age-depth models were obtained by applying the Constant Rate of Supply (CRS) model developed by Appleby & Oldfield, 1978^14^, which is the most approached model for determining chronologies in recently deposited sediments (< 200 years). The CRS model considers the dilution of ^210^Pb concentration by accelerated sedimentation rates, as such, the unsupported ^210^Pb fraction will be inversely proportional to the mass accumulation rate, while also assuming a constant fallout of ^210^Pb, and subsequently, a constant rate of its supply to the sediment surface^14,15^. The age calculation methods for the CRS model are extensively described by Appleby & Oldfield, 1978^14^, the sedimentation rate (SR) was obtained from the slope of the linear regression of depth plotted against the logarithm of the unsupported ^210^Pb and the decay constant.

## Results and discussions

**Figure 2:**
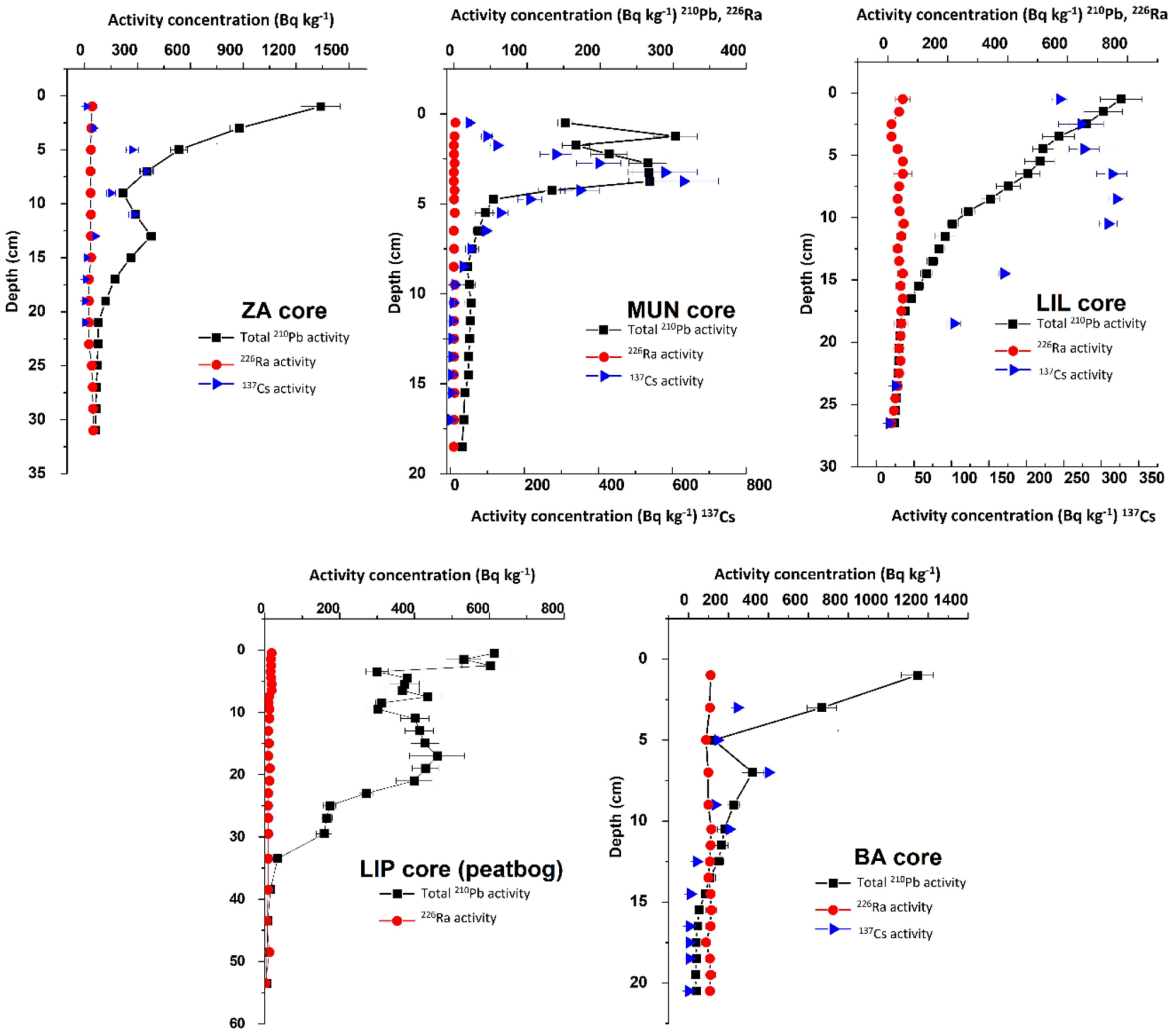
Radionuclide-specific activities (Bq kg^−1^) plotted against depth (cm) for the investigated lakes and peat bog columns.

The radionuclide specific activities in the sediment (Fig. 2) are in agreement with the average values recorded in lakes throughout the literature, while in the LIP peat bog the negligible values of ^226^Ra (mean 3.9 ± 0.20 Bq yr^−1^) are indicative of an exclusively atmospheric supplied mineral material (ombrotrophic bog). The two peaks of ^137^Cs activities for 1986 and 1960s, used as time markers, are present in the sediment and could be identified. The variations in the total activity budget are regarded as normal and are caused by differences in the local meteorological conditions across the areas during the passage of the radioactive cloud. These factors control the predominant deposition mechanism (wet or dry), which in turn will render different levels of ^137^Cs contamination on the soil surface of the catchment area.

**Figure 3:**
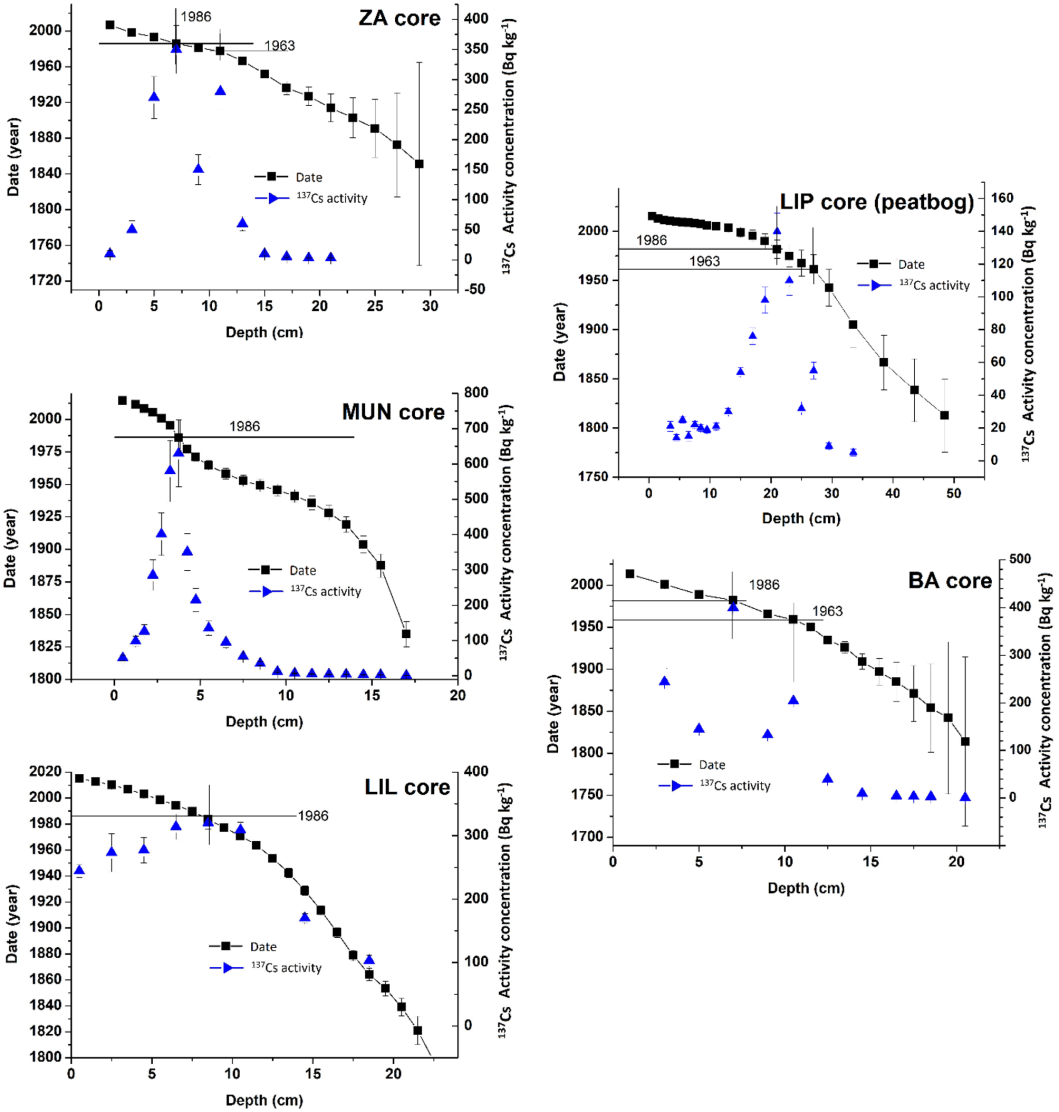
Age-depth chronologies following the CRS model for the investigated lakes and peat bog columns.

The high-resolution chronologies were constructed based on the CRS model, and the age calculations were performed for each layer, from the ^210^Pb excess (or unsupported) fraction results (Fig. 3). The ^210^Pb activities yielded a relatively well-defined exponential decrease with depth, with a few variations that may be indicative of fluctuations in the intensity of the sedimentation rates. The relatively low dating errors (errors increasing with age, generally under 5 years for 1950) ensured the comparability of the sedimentation rates across the investigated lakes. ^137^Cs was employed as an alternative time-marker to validate the obtained ages, and the 1986 Chornobyl maximum activity depth horizon overlaps the ^210^Pb CRS model age results, thus reassuring the quality of the obtained chronologies.

Information on regional climate variability throughout the studied period is critical to understanding the sedimentation dynamics of the lakes, as well as to distinguish the anthropic factors from the natural ones. The meteorological data in the region of Southern Carpathians is provided by 13 well-distributed stations, managed by the Romanian National Meteorological Administration (NMA). From 1961 to 2018 the regional climate of the study area exhibited significant changes, with intensified heat stress and milder thermal winter conditions, especially after 1990^16^.A general decrease in freezing days is observed, with more intense trends at high elevations (− 5.2 days decade^−1^), as well as a prevalent increase of absolute maximum and minimum temperatures throughout the region^16^. The duration of warm spells increased significantly after 1980 and drastically after 2000. The average warmest daily temperature index increase is estimated at 0.47 °C decade^−1^^16^, with the years 2000, 2007, and 2012 registering the highest temperatures. The increase in consecutive dry days is positive for all stations, yielding a regional rise from 0.26 to 1.15 days decade^−1^^16^, with stronger trends in the lower part of the alpine belt (~ 2000 m a.s.l.). Although over the period between 1961 and 2018, the area is becoming increasingly drier, with a decrease in total precipitations recorded by more than half of the meteorological stations, there is a large increase in precipitation intensity at the Bâlea lake station, holding the record for greatest 1-, 3-, and 5-day precipitation values of 195.6 mm, 368.6 mm, and 133.3 mm respectively. An increase in the extreme precipitation index of the greatest 1-day precipitation is dominantly positive at 62% of the stations, with the total precipitations in wet days showing a visible decrease since 1984, and an increase in positive anomalies after 1994^16^. The drying trends are stronger in the areas exhibiting positive temperature anomalies, indicating a combined dry-warm climate change signal in the region.

**Figure 4:**
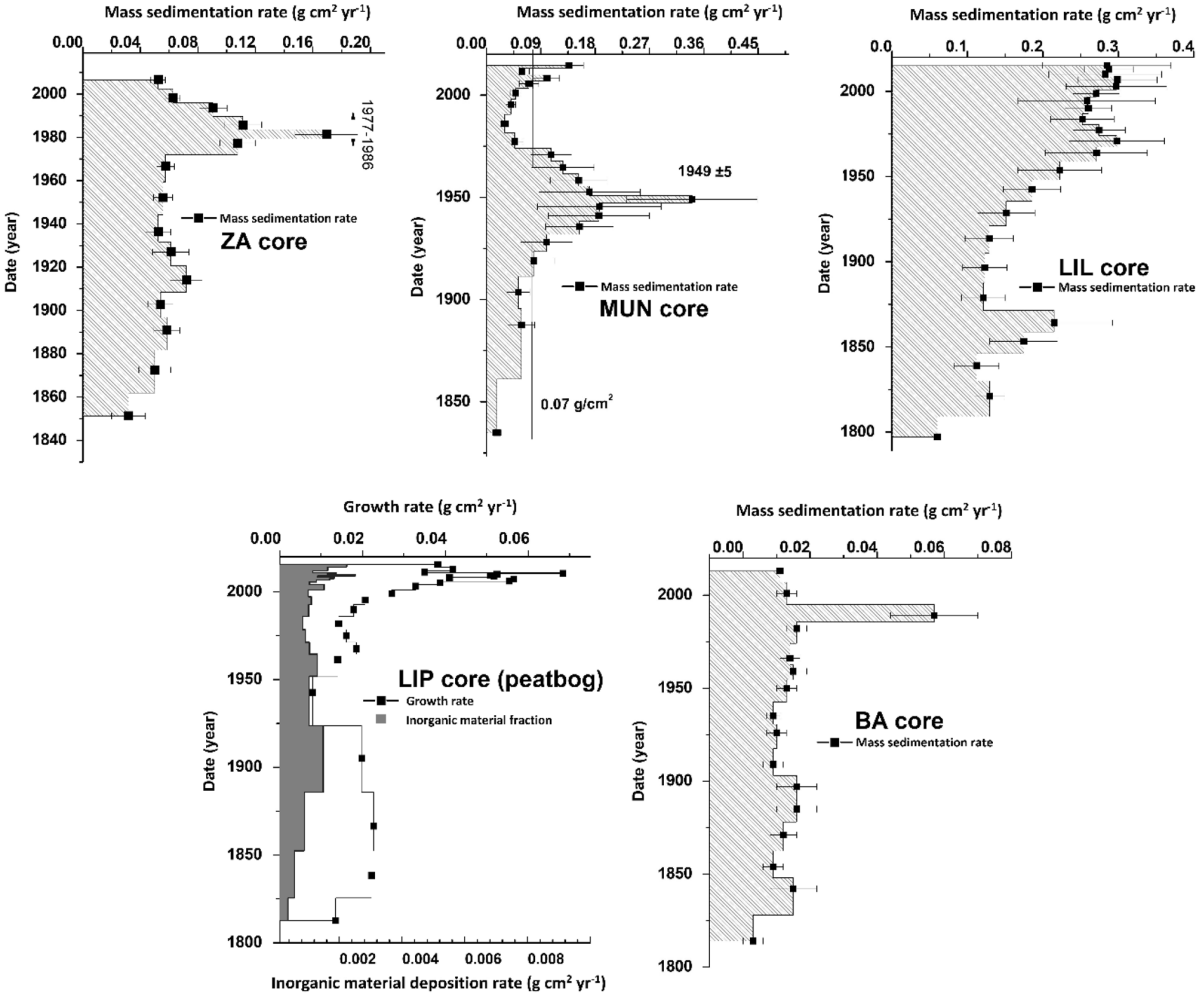
Time-integrated mass sedimentation rates for the investigated lakes and peat bog columns.

For the investigated period, Zănoaga (ZA) lake mass sedimentation rate (Fig. 4) varied between 0.031 ± 0.011 and 0.169 ± 0.021 g cm^2^ yr^−1^, with a mean value of 0.072 ± 0.034 g cm^2^ yr^−1^. A high sedimentation event occurred in the catchment area that corresponds to the 1977–1986 period (mean SR of 0.129 ± 0.034 g cm^2^ yr^−1^), with a peak in 1981, where the SR tripled, reaching the maximum of 0.169 ± 0.021 g cm^2^ yr^−1^. Due to the relatively isolated position, and insufficient information in the literature regarding the ZA lake, it is difficult to correlate the high sedimentation event observed with certain anthropic interventions in the area. Land cover change is a conceivable explanation for the increased mass SR results, although natural factors may be engaged.

The Muntinului (MUN) lake mass SR (Fig. 4) presented the highest variance, between 0.016 ± 0.006 and 0.340 ± 0.108 g cm^2^ yr^−1^, with a mean of 0.107 ± 0.074 g cm^2^ yr^−1^. From 1936 until 1959, a high sedimentation period is visible in Fig. 4, during which the mean SR reached 0.198 ± 0.071 g cm^2^ yr^−1^, tripled compared to the previous period. The proximal position of the lake relative to the Transalpina DN67C road (the highest national road in Romania) may be associated with the high sedimentation period, as the road was rebuilt during 1934–1939 and further rehabilitated during the Second World War. The soil displacement associated with the construction works in the area may be a primary cause for the increase in sedimentation trends during the 1936–1959 period. The recent increasing trend in sedimentation since 1970 is corresponding with the decrease in forest area associated with hydro-technical constructions in the region^17^.

The SR in Bâlea (BA) lake (Fig. 4) was the lowest of the investigated cores, varying between 0.003 ± 0.002 and 0.057 ± 0.012 g cm^2^ yr^−1^, with a mean value of 0.014 ± 0.018 g cm^2^ yr^−1^. A high sedimentation event that yielded the maximum SR for the core (0.057 ± 0.012 g cm^2^ yr^−1^, almost 5 times higher compared to the mean value) can be attributed to the year 1989. This sedimentation event coincides with the 1988 avalanche (resulting in a large snow deposit close to the lake) in the area^18^, while in the same year, the maximum precipitation was recorded by Bâlea Lac meteorological station indicating 386.3 mm for five consecutive days^16^. The lake does not record any substantial sedimentation rates from 1969 to 1974, during the Transfăgărășan DN7C national road construction works, indicating minimal effects on SR.

The Latoriței (LIL) lake mass SRs (Fig. 4) varied between 0.060 ± 0.017 and 0.298 ± 0.052 g cm^2^ yr^−1^, with a mean value of 0.215 ± 0.074 g cm^2^ yr^−1^. Contrary to the previous lake cores, the LIL sedimentation rate trend over the past 150 years showed a rather continuous and constant increase, with substantial sedimentation shifts largely absent. The pattern exhibited by LIL mass SR is indicative of climate variability, as highlighted by Pont et al., 2002^9^, naturally-induced variations in sedimentation rates follow an elongated curve that stretches over a longer period, rather than the specific high-intensity short interval sedimentation peaks associated with significant anthropic interventions. The Transalpina DN67C road constructions (1934–1939) did not induce a visible shift in sedimentation rate as it did for MUN lake, this can be explained by the difference in distance from the road (200 m from MUN, while almost 4 km for LIL).

The Latoriței peat bog core (LIP) (Fig. 4) revealed growth rates ranging between 0.011 ± 0.008 and 0.078 ± 0.058 g cm^2^ yr^−1^, with a mean value of 0.037 ± 0.018 g cm^2^ yr^−1^. A similar rise in the trend is apparent in the plotted growth rate against age, which is justified by the accelerated global climatic changes, inducing higher mean temperatures and a larger growing season, further providing suitable conditions for peat growth. This pattern and subsequent correlations have been observed and reinforced by a multitude of authors throughout the scientific literature^19–21^.

Throughout the LIL and LIP investigated cores, similar depositional patterns are recognized, indicating similar contributing factors to the variations in the exhibited sedimentation rates. Furthermore, since the investigated peat bog is regarded as a ”raised” *Sphagnum*-dominated, ombrotrophic ecosystem, the growth rate is solely controlled by atmospheric precipitations, representing its exclusive source of nutrients and water^22^. Based on the previous statements, it can be confidently assumed that the controlling factor of the depositional variations recorded in the LIL and LIP cores is related to climate variability (temperature and precipitation). This affirmation is further strengthened by the spatial proximity of the two ecosystems (but different watersheds), which renders highly improbable any common anthropic cause for the observed events.

**Figure 5:**
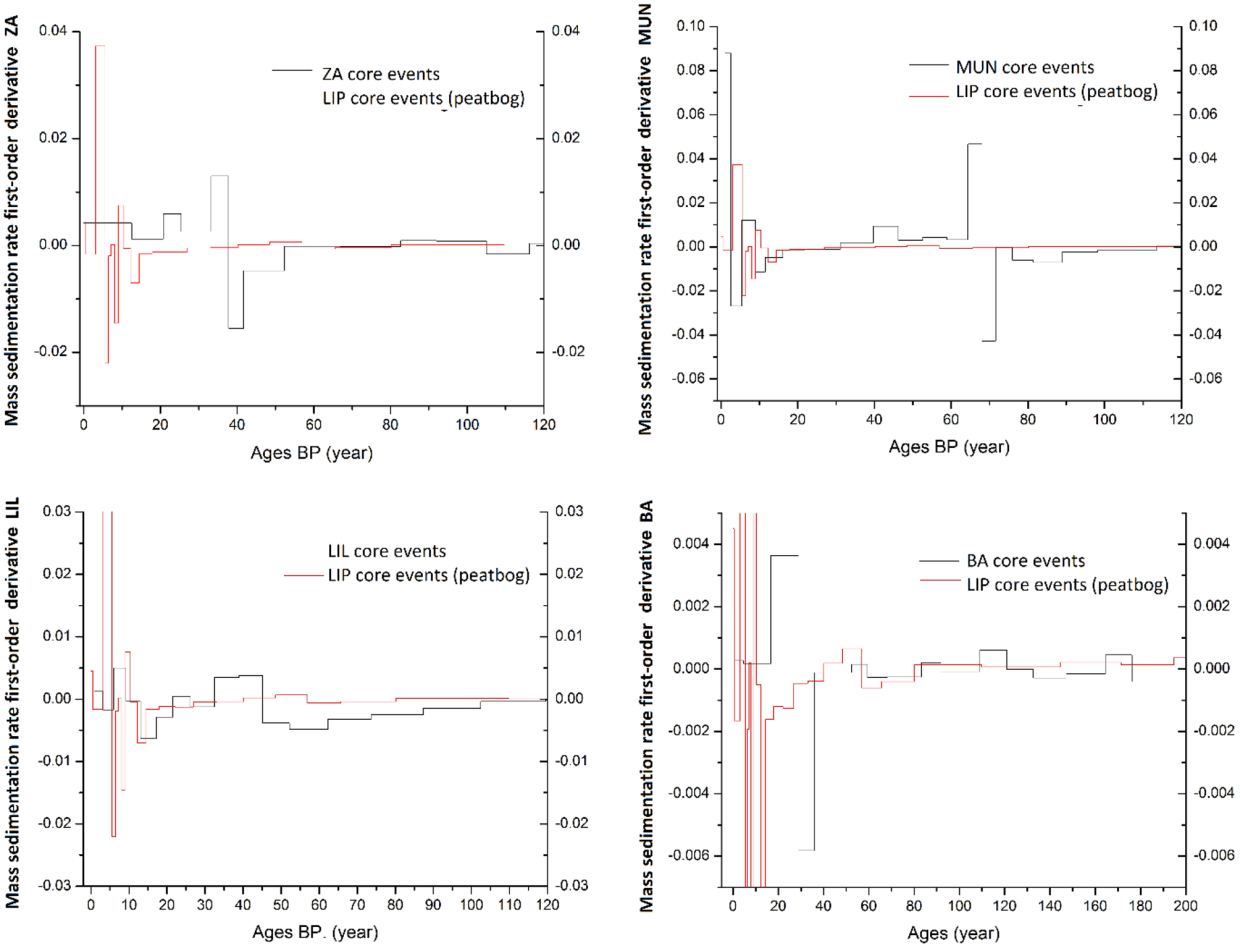
First-order derivative equation describing the acceleration of the change in sedimentation rate for each lake compared to the LIP peat bog, plotted against time.

The second motivation of the present study was to identify the cause, either anthropic or natural, accounting for the variability in sedimentation rates, by comparing the results with a baseline. Based on the above-stated affirmations, the LIP peat bog core sedimentation trend was regarded as suitable for baseline. A first-order derivative equation was employed to perform an intercomparison of the change acceleration in mass sedimentation rate, as a function of time, between the investigated lake cores and LIP peat core. The equation was meant to provide a more reliable term of comparison between the obtained sedimentation trends. Figure 5 describes the results obtained by comparing the first-order derivative equation outputs of the LIP peat bog core and the studied high-altitude lake results.

In each instance, a rise in the acceleration of mass sedimentation rate is observed for the recent ages (previous 20 to 30 years). The timeframe of these intense changes overlaps with the period of the most pronounced climatic changes. A multitude of factors, such as rainfall frequency and intensity, multiyear droughts, and temperatures have a direct impact on runoff and sedimentation rates, by increasing flood events and reducing the vegetation cover^23^. These extreme hydroclimatic events have increased in frequency and intensity under contemporary climate changes^24,25^ and can be related to recent temporal trends of sediment accumulation^26^.

Compared to LIP, BA lake manifests two high-acceleration occurrences in the sedimentation rate, that coincides with the same period of 1989, previously discussed, during which the sedimentation rate increased by 3.6 times and immediately declined by 4.36 times. This event coincides with the avalanche and the maximum precipitation amount recorded in 1988 at this site^16,18^. For the rest of the investigated period, there are no significant differences in the acceleration of the SRs, which matches the baseline trend within uncertainties.

The MUN lake has three such occurrences, two corresponding to the 1936–1959 period, during which the SR reached its peak value (3.42 times higher than the previous period). Although this variation in the mass SR stretches over a longer period, it is highly improbable to be associated with climatic variations, as both MUN and LIP are located within the same geographical area, with a distance of under 4 km between the sites. For this reason, the absence of a similar signature in the LIP or LIL acceleration trends renders the exclusion of a natural cause and further highlights the applicability and functionality of this approach in the investigation of the mass SRs causes. Another rise in the acceleration of the mass SR of MUN is visible for the recent period, starting from 2015, during which the mass SR value more than doubled compared to 2012, this difference is indicative of an anthropic cause, as no similarities can be identified in the baseline trend of LIP. The most probable cause of this event is the development of tourism in the area in the recent period. Analyzing the orthophotos of the site from 2011 to 2023, it is clear that a tourist spot (several small buildings and a viewpoint) was constructed from 2011 to 2015 next to the lake, intensifying the anthropic influence in the area.

For ZA lake, a high increase in SR event followed by a sharp decrease is identified, corresponding to the 1977–1986 period, during which the sedimentation rate reached a triple value compared to the mean. Although this is regarded as an intense sedimentation event, the most plausible cause could be related to natural changes in the catchment. From the first-order derivative equation, we can state that compared to the LIP peat bog, there are visible deviations, however not as intense as MUN. Moreover, the location of the ZA lake in a national park with no significant infrastructure constructed in its proximity proves that an anthropic influence could be very limited and highly improbable.

The presented approach is intended to provide an initial classification of the variation in mass sedimentation events based on their deviation from the baseline trend. Particularly in the absence of a documented history of the lake catchment, this method can supply relevant information that can further motivate the use of supplementary proxies (e.g. elemental, grain size analysis) to validate the driving factor of the SR fluctuation. A large proportion of the high sedimentation events may result from a combined effect of both anthropic and climatic nature. For instance, sediment transport from overcultivated lands can be enhanced by severe flood and drought events, resulting in an aggravation of the lake sedimentation^3^, with the same effect being achieved by land-use change, which was linked with temperature increase^23^. A particular challenge linked with this approach is represented by the difficulties associated with locating an anthropically-unperturbed lake/peat bog in the study area, that can constitute the baseline for the climatic variations. Furthermore, a well-defined high-resolution chronology of the supposed sediment core is essential for ensuring high-quality of results.

## Conclusions

The present study investigated the temporal variation in sedimentation rate in four high-altitude lakes and a peat bog located in Southern Carpathians, Romania. The high-resolution chronologies were constructed following the ^210^Pb dating method and the Constant Rate of Supply (CRS) model. ^137^Cs activity peaks overlapped the age-depth models, further validating the obtained results. The mass accumulation rate results highlighted intense, generally short-term sedimentation events in lakes, except for LIL which followed a continuous rising trend. LIP peat bog growth rate results indicated exceeding similarities with LIL, thus indicating a preponderantly climatic-driven variation in the sedimentation trend. A first-order derivative equation was employed to allow for the comparison between the change acceleration of sedimentation rates exhibited by the investigated lakes and a baseline represented by the peat bog core. The results underlined significant differences between the high mass sedimentation events identified in MUN, BA, and ZG lake cores and the baseline, thus highlighting presumably anthropically attributed events. The main anthropic drivers of the sedimentation rate in the studied region were road construction and rehabilitation, reduction in the forest cover, as well as increased tourism in the catchment area. This approach was intended to deliver preliminary information regarding the nature of the dissimilarities in sedimentation events that enable further considerations of multi-proxy analyses for validation of the assumptions.

## Data Availability

All data generated or analyzed during this study are included in this published article.
